# Book Reviews

**Published:** 1827-07

**Authors:** 


### CRITICAL ANALYSES.

Quæ laudanda forent, et quæ culpanda, vicissim

Illa, prius, cretâ; mox hæc, carbone, *notamus*.—PERSIUS.

*Commentaries on some of the more important of the Diseases of Females.* In three Parts. By MARSHALL HALL, M.D. F.R.S.E. &c. &c.—8vo. pp. xv. 376; with eight coloured Plates. Longman and Co. London, 1827.

DR. HALL having, upon more than one occasion, distinguished himself as an able and useful contributor to our information upon different medical subjects, we naturally approach the present more matured produce of his labours, with the expectation of deriving information ourselves, and of being able to extract from the volume matter that will prove interesting to our readers. The work treats of some of those forms of the diseases of females which, from affecting the system in general, or some important cavity or organ, fall under the cognisance of the physician. The reader is informed that the author has, upon the present occasion, not only embodied in one work his former observations,* but that he has entered more fully into the investigation of the subjects upon which he has before treated, from a conviction that a renewed inquiry into their diagnosis, or essential difference under a similarity of appearances, and into their nature, pathology, and treatment, will lead to many important and beneficial results.

Although many years have been devoted to the consideration of the diseases he now treats of, the author does not presume to suppose that he has exhausted the subject. He is perfectly aware that the inquiry still affords great scope for further investigation. It has been thought advisable to divide these commentaries into three parts.

"The first part will comprise those morbid affections which are principally incident to female youth. They chiefly involve a state of derangement of the general health, a morbid condition of the functions of the uterine system and of the mammæ, and, not unfrequently, a defective development of the form during growth.

"The second part will comprehend those morbid affections which supervene during pregnancy, parturition, and the puerperal

* See an Essay on Disorders of the Digestive Organs and of the General Health, first published in 1818; an Essay on a Serious Morbid Affection, occurring after Delivery, Miscarriage, &c. published in 1820; and Medical Essays on the Effects of Intestinal Irritation, and of Loss of Blood, and on Exhaustion and Sinking from various Causes, published in 1826. See also the Medico-Chirurgical Transactions, vol. xiii. p. 121 and p. 189.

state. They consist principally in affections of the general system, or of some important cavity or viscus, and, arising from very various or even opposite causes, necessarily vary in their nature and treatment. It may be observed, in general, that the disorders of early pregnancy do not arise from the condition or situation of the uterus itself, but from its sympathies with distant organs; but, in the later periods of pregnancy, from the irritation or pressure of the gravid uterus, inducing fulness of the blood-vessels, or other affection, within the head, or œdema of the extremities. During parturition, the patient is exposed to the effects of uterine irritation or pressure in a more especial manner; but afterwards to a state of great comparative inanition, both by the removal of a long-continued source of pressure, and generally by the occurrence of hemorrhagy. In the puerperal state, the patient is variously liable to suffer from milk-fever and from other kinds of fever, from inflammation, from intestinal irritation, and from loss of blood; and from two or more of these causes variously combined and co-operating together.

"From this slight sketch of the second division of the class of female diseases, it will be obvious that much, very much, still remains to be done before the investigation into their nature and treatment can be said to be at all complete.

"The third part will comprehend those affections which are incident to the middle and later periods of life: they are chiefly constitutional, and consist in derangement of the general health, or arise from undue lactation, from menorrhagia or leucorrhœa, or from the circumstances of the two climacteric periods, of the cessation of the catamenia, and of the decline or sinking of the vital powers in advanced years." (P. vii.)

Chapter I. *Of the disorders incident to female youth in general.*—Many circumstances concur to render the disorders of female youth different from those of the male sex; but chiefly peculiarity of constitution, and that important change which is at this period effected in the uterine system. "The peculiarity of constitution existing in the female sex, and modifying its disorders, appears to consist principally in a greater development of the capillary vessels, and in a greater susceptibility of the nervous system, than are observed in the male sex." The accuracy of the latter part of this statement is doubtless fully impressed upon every practitioner who has had opportunities of watching the progress of female diseases, more particularly at the approach to womanhood, and who has not omitted to take advantage of his experience. With respect to any peculiar "and greater development of the capillary vessels," perhaps some doubts may exist, and some proofs may be required that this is not an hypothetical opinion rather than a fact established by observation.

Dr. Hall observes, "that derangements in the return and flow of the catamenia, after these have once appeared, are also most frequently the *effect* of some disorder of the general health; so that a continued and watchful attention to this point is still essential. In other cases, an undue suppression or flow of the catamenia, or a leucorrhœal discharge, is the *cause* of derangement of the general health."

Our experience in the nature and management of female complaints has been considerable, and we are inclined to believe, from attentive observation and much reflection, that in by far the greater number of cases in which the health is deranged, and the catamenial discharge at the same time interrupted, we must view the latter circumstance as an effect, and not as the cause, of the constitutional suffering. This is but an iteration of Dr. Hall's opinions; but we have had such frequent proofs of the mischief inflicted in the ordinary practice of the day, by the administration of "forcing medicines," without reference to the general health of the patient, that we could not pass over the subject without offering a remark upon it.

The evils arising from a constipated state of the bowels in young females, are properly considered by Dr. Hall as the most frequent forerunners of various and more serious ailments.

"It is to be presumed, too, that, with the state of the alimentary canal, the organs which contribute to digestion, as the liver, pancreas, &c. are proportionately deranged in their functions; digestion is variously disordered; the contents of the intestinal tube become morbid; and these again react upon each other reciprocally. Nutrition is also frequently impaired, and there is, in consequence, a certain degree of loss of flesh; and it is a point which I have ascertained by repeated observation, that, after a certain duration of a disordered state of the digestion and of the general health, and associated with a peculiar appearance of the tongue, which I have termed the lobulated, there is frequently simple enlargement of the liver. Upon the subject of affections of the liver, however, I would earnestly renew a caution which I gave some years ago, not to consider every icterode hue in the complexion or general surface as denoting disease, or even disorder of this organ. This state of the cutaneous surface is frequently the effect of a loaded condition and impaired function of the alimentary canal; and it is, in various instances, an affection of each of the cutaneous textures, or of the cutaneous circulation, altogether independent of any tinge of bile." (P. 8.)

This observation of Dr. Hall's is of much importance. Many a patient is *mercurialised* whose countenance exhibits the appearance he refers to, and which is erroneously considered an infallible indication of hepatic disease or derangement.

To preserve the health of the young female, the necessity of frequent exercise is strongly insisted upon. We really, however, are not aware that the custom of closely confining young persons, of either sex, to sedentary employments is now so general as the author appears to imagine. In all the families under our own inspection, there appears to be as much attention to invigorate the body by well-timed exercise as there is to improve the mind by a necessary attention to intellectual studies.

Chap. II. *Of disorder of the general health in its more acute form.*—

"The general character and symptoms of this disorder are very distinct and characteristic; but its complications are extremely various, and sometimes predominate over the other symptoms, and resemble some topical affections so much, that it is of the utmost moment to institute a correct diagnosis between them. With the view of assisting in the distinction of this morbid affection in future, it will be my object, first, accurately to detail the symptoms which characterise the complaint in general; and, in the second place, to enumerate and describe those particular complications which are apt, in certain instances, to engross the attention of the patient and of her friends, and even to occasion some degree of embarrassment to the physician.

"This affection, even in its more acute form, comes on insidiously, and the patient becomes gradually and insensibly incapacitated for exertion either of mind or body. This state of unconscious disorder, perhaps, endures for many months before it attracts the serious attention of the patient or her friends; and when a medical opinion is taken, it is usually sufficiently characterised by a general feeling of weakness, with tremor, headache, or vertigo, fluttering, faintishness, tendency to perspiration on the least exertion or surprise, susceptibility to hurry and agitation, weariness, aching, and loss of flesh; and with these symptoms there are peculiar states of the countenance, of the tongue and internal mouth, of the general surface, and of the evacuations, which I now proceed to describe. I would first observe, however, that, although the accession of this morbid affection is usually slow and insidious, it is occasionally more rapid, being induced by the occurrence of some other indisposition, or of a fall or other accident." (P. 15.)

The peculiar state of the tongue which exists in this general derangement of the health, is represented in Plate I. fig. 1, 2, and 3. In this affection, the patient is generally annoyed by much nervous tremor. She feels unaccountably feeble and weary, and suffers from a sense of aching after slight exertion. There is an early, but very slow and gradual ema-

Fiddle faddle ciation, "and it is interesting to remark, by weighing the patient, first, the still continued progress of the loss of flesh for a time; and then the cessation, and, lastly, the restoration from this fearful morbid process, on the institution of a correct mode of treatment." (P. 20.)

There is almost universally a peculiar sense of fluttering about the heart and pit of the stomach; the pulse is variable; the state of the digestive function is also uncertain.

"Doubtless the uterine discharges, if carefully examined, would be found to have participated in the general disorder; but it is only in the more protracted cases of this morbid affection that they are so obviously changed in their appearance, quantity, duration, and returns, as to excite the attention of the patient.

"It is extremely important to remark, that the condition of the countenance, of the tongue and internal mouth, and of the general surface, is peculiarly constant and uniform in almost every case, and at every period of this disorder; whilst the other symptoms are as characteristically inconstant and variable. It is, therefore, by a recurrence to the former that we are frequently enabled to establish a certain diagnosis, in cases which might otherwise be doubtful; and it is by the very variable character of the latter even that we derive a further source of diagnosis." (P. 23.)

The predominating symptom is not the same in different cases, nor even in the same case at different periods, but it is sometimes an affection of the head, sometimes of the heart, &c. We believe that no practitioner, with a moderate share of experience, will fail to recognise the accuracy of the description given by the author of this disorder of the general health of young females, which is not attended by symptoms of sufficient constancy or regularity to enter into our nosological arrangements. We could, if it were not our object to confine ourselves to the notice of Dr. Hall's book, rather than to give an essay upon the subjects of which he treats, detail many cases of considerable interest from our own practice, in which, at different periods, and within a short space of time, the patient has appeared to labour under organic disease of almost every organ in the body. We do not, however, remember any case of this kind, in which general hysterical symptoms of a well-marked nature did not alternate with the varying local affections.

Having attended to the general character of the complications observed in the more acute form of disorder of the general health, the author proceeds to describe each of these complications separately, and to point out the diagnosis between them and idiopathic diseases of the same organs. In such cases caution is of course necessary. That which was at first a mere functional complication may, in the sequel, become organic disease. The patient must therefore be attentively watched.

"During the whole course of this disorder, the patient is peculiarly liable to be affected with fluttering, irregular action, or violent palpitation of the heart, or with syncope. The affection is distinguished by observing the concurrent appearances and symptoms, by carefully observing the effects of remedies, and the state of the symptoms from day to day,—but especially the influence of bodily exertion, when the patient is least under the influence of these painful feelings. Organic disease of the heart is ever present, and ever exasperated by exertion; the symptomatic affection is apt to subside, and then exertion may be borne without inducing the sufferings experienced in the former case.

"Hæmatemesis and melæna are not unfrequent complications of this form of disorder, and especially in females. Hæmatemesis is immediately obvious, but melæna is ascertained only by accident, and frequently occurs unknown and unsuspected by the patient. They are frequently conjoined in the same case, and appear to be a similar affection of different parts of the alimentary canal.

"Disorder of the general health is sometimes accompanied by the most decided jaundice.

"In the course of this disorder there is frequently pain, more or less severe, more or less continued, and in such different forms and situations as to resemble, in different cases, attacks of gall-stones, inflammation of the pleura, liver, spleen, kidney, or of the intestine or peritoneum; and there is sometimes a sensible hardness, or tumor, which appears to consist in a loaded state of the intestines. This occurrence is particularly apt to take place in females.

"There is sometimes in this disorder symptoms of affection of the bladder, probably arising from a loaded or irritable state of the rectum." (P. 28.)

The first object in the treatment of this morbid affection is to regulate the bowels. Care, however, must be taken that purgatives are not employed to such an extent as to produce irritation and distention in the alimentary canal, and exhaustion and nervousness in the general system, more distressing than the original disorder. Mercurial purgatives are seldom required, in the opinion of Dr. Hall. The diet should be mild, light, and nutritious; and the mind of the patient must, if possible, be kept in a tranquil state. Even with the most judicious mode of treatment, a rapid cure is not to be expected.

In our endeavours to relieve the affection of the head which is so common an attendant of the general disturbance above described, we must carefully bear in mind "that the very sensations of affection of the head, which are apt to be induced by a disordered state of the bowels and of the general health, are kept up, and perhaps aggravated, by undue depletion. Inanition produced by too active purging, or by the too considerable abstraction of blood, is alike apt to be attended by headache, vertigo, or a sense of weight or of confusion. In this case, eating frequently relieves the affection of the head."

Chap. III. *Of disorder of the general health, in its more protracted form.*—After an uncertain continuance of general indisposition, the patient approaches a cachectic state, which must be combated by the occasional exhibition of cordial purgatives, tonics, fresh air, and other obvious means of restoring the broken powers of the constitution.

Chap. IV. *Of Chlorosis.*—The constitutional derangement treated of in the preceding chapter appears to us very nearly allied to chlorosis, and Dr. Hall himself—

"Thinks the form of disorder of the general health, about to be described, differs from those treated of in the two preceding chapters, chiefly in the peculiar character of the constitution of the patient. It principally occurs in female youth, but not unfrequently in married persons; and it is not entirely unknown in the young and delicate of the male sex. It chiefly affects persons of what may be justly termed the lymphatic temperament; whilst those of a different temperament are most liable to that form of disorder to be noticed in the ensuing chapter.

"I have observed a morbid affection very similar to this in persons of the middle age of both sexes; and I have had occasion to remark, that in these there had been either hemorrhagy or some other cause of debility or exhaustion." (P. 51.)

Dr. Hall is persuaded that there is seldom any truth in the conjecture that sexual excitement is the cause of this form of disease. The result of our own experience would induce us to give a contrary opinion. Many such cases have fallen under our care, and in most of them it has appeared that the genital system was in a high state of excitation, however anxiously the patient might endeavour to conceal the fact.* The appearances of the chlorotic countenance are accurately pourtrayed in the fourth plate. The state of the tongue in the different stages of chlorosis is also well represented in Figures 7, 8, and 9 of Plate 3d.

Dr. Hall despairs of giving any thing accurate or specific

* On this point we must take leave to differ with the reviewer—in well marked cases of chlorosis, there has always appeared to us to be a general torpor involving the genital system, quite as much as any other organs.—Ed.

in regard to the pathology of this and other forms of disorder of the general health.

"There appears to me not to be a system, an organ, a texture, or even a fluid in the animal economy, which does not suffer in different instances of this multiform disorder. It has already been shown that the complications of the more acute form of disorder of the general health differ totally from those of the more protracted, both in their various seats and in their nature; the former affecting the more vital organs, the latter the superficial textures. A similar remark equally applies to the form of disorder of the general health under consideration." (P. 60.)

We agree with Dr. Hall that the first cause of this affection, in most instances, is to be found in some derangement of the bowels, which, we may add, is in young females frequently neglected, until the supervention of other symptoms which are considered more worthy of attention. The practitioner is again very properly cautioned not heedlessly to have recourse to blood-letting and blistering, for the purpose of relieving the apparent local diseases with which the general affection is not unfrequently complicated. We have seen several instances similar to that referred to by the author, in which the patient has been at different times treated for visceral inflammation without effect, and in which the general health has been promptly and perfectly established by a more appropriate plan.

"The diagnosis is founded upon the state of the countenance, of the tongue, of the general surface, of the bowels, and of the catamenia, the multitude and variety of the other symptoms, the variable history of the case, and perhaps the suddenness and repetition of the attack, and the effects of remedies. The only difficulty exists when some topical inflammation comes on in a patient previously affected with chlorosis. Even in this case the disease has assumed a more settled and definite form, instead of the varying and complicated character of chlorosis, and may then be distinguished by a careful examination.

"These observations strictly apply to the diagnosis of chlorosis with pain of the head, from inflammation of the brain or its membranes. In the latter disease there are not the characteristic appearances and symptoms of chlorosis, as observed in the countenance, tongue, general surface, and general symptoms; but there are, on the contrary, the peculiar and definite symptoms and history of inflammation of the encephalon, which it would be out of place to mention here.

"The cough and dyspnœa, the palpitation of the heart, the pain of the side, and the pain and tenderness of the abdomen, are to be distinguished from inflammation within the chest or the abdomen, in the same manner, by comparing the general and local characteristics of chlorosis with those of each of these diseases, and by ascertaining the history, and observing the effects of remedies.

"The pains of the side, or of the abdomen, so apt to occur as complications of chlorosis, are to be distinguished from pleurisy or peritonitis by the same recurrence to the state of the complexion, tongue, and general surface, and to the other symptoms, and by their own peculiar character: these pains, for instance, are less constant, both in their situation and duration, than those of an inflammatory nature; and, though sometimes aggravated by a deep inspiration, are not invariably so, especially on repeating the inspiration a third or a fourth time. The accession of pain of the side in chlorosis is apt to be sudden; the side affected is sometimes changed; the degree of pain sometimes extremely severe, at others less so; and there is more expression of pain than is permitted by the pain of inflammation, which represses the movements of respiration, implied in the loud expression of pain." (P. 64.)

The treatment of this form of disorder of the general health must be begun by due evacuation of the bowels. The use of mercurials and active purgatives requires caution, and the addition of mild cordial remedies is still more necessary than in that form of disorder described in the two preceding chapters. As aperients, Dr. Hall prefers aloes and rhubarb.

"When the bowels have, by these means, been fully but gently regulated for some time, different preparations of iron, but especially the sulphate, become specific in this disorder,—gradually restoring the complexion, the general surface, and the uterine discharges, to their healthy state. The condition of the complexion, and of the catamenia, constitutes the true indication for the employment of these remedies, and of their beneficial effects when these begin to be displayed. I do not mean to confine the use of chalybeates to the cases in which the prolabia are exanguious and the catamenia are pale, but I can affirm that their efficacy is most unequivocal in these cases." (P. 66.)

For the local pains, cupping may be tried; "but it is extremely important, in chlorosis, to avoid taking blood as much as possible."

Dr. Hall does not mention the good effects of horse exercise. In several instances we have found it of decided benefit; and, where the strength of the patient is not too far reduced, we should recommend it with much confidence as an auxiliary to the general plan of treatment.

The sixth chapter gives a very brief sketch of the more prominent forms of Hysteria.

The eighth chapter contains some interesting remarks upon the derangement of the general health observed in females returned from India. The morbid influence of the climate of India upon the general system, and upon the liver and alimentary canal, is well known; but it appears to Dr. Hall "that the effects of a residence in India upon the functions of the uterus have not received the degree of attention which they demand." Mr. C. M. Clarke mentions the interesting case of a European child, who went to the West Indies at the ago of six years, in whom menstruation took place at the ninth year, and continued to recur regularly for three months; but the child then returning to a more temperate climate, the secretion ceased, and had not returned when the child was twelve years old. "Change of climate is generally of the utmost service in restraining the uterine discharges; but the effects of the previous drain and losses of blood do not so soon cease, and the patient presents all the symptoms of exhaustion, either in that form which is attended with reaction, or in that in which the symptoms of reaction do not manifest themselves. In addition to exhaustion, there are also very frequently the effects of intestinal irritation, the bowels being extremely apt to become confined." (P. 102.)

Chapter IX. *Of the diagnosis and symptoms of some local inflammatory diseases.*—From the observations already made, it must be evident that, without considerable caution, blood-letting and other debilitating modes of treatment will be had recourse to in many of the general derangements of females, when such a plan is not only unnecessary, but decidedly prejudicial. The following observations merit very particular attention.

"Whenever there are the appearances of complexion, tongue, and general surface, which have been described as obtaining in the different forms of disorder of the general health, the presumption will be that any local affection is only a symptomatic complication of that disorder. This presumption is strengthened if there be an entire absence of any external cause of the local affection. But it is to be carefully observed that it is still only a presumption, and that there may be a concurrence of idiopathic local disease with disorder of the general health; or that that which was symptomatic at one period may become actual disease in its course, especially in the more acute form of disorder of the general health described in the second chapter of this work; for this very rarely, perhaps never, occurs in the other forms of this disorder.

"If there be many of the general symptoms of disorder of the general health which affect the head, the heart, the breathing, the nervous and the muscular systems, &c. there is a still further, and I think a still stronger, presumption that any predominant topical affection is symptomatic; for I have repeatedly observed that idiopathic disease frequently subdues these symptoms, even when they had previously existed, and gives a definitiveness to the affection which it had not before, and which returns only when the idiopathic disease is subdued." (P. 113.)

For some years our attention has been very particularly devoted to hysterical affections, in all their variable forms, and we perfectly agree with Dr. Hall that, although hysteria cannot be considered incompatible with idiopathic inflammatory disease, attacks or symptoms of the former very rarely occur with the active inflammation of a vital organ. But we hesitate to agree in the opinion, "that in the greater number of the cases of the occurrence of hysteria after blood-letting, that the original complaint was not inflammation, but disorder of the general health with some local complication." Females of a weak and excitable constitution may labour under acute visceral inflammation, for which blood-letting becomes absolutely necessary, although not to such an extent as in those of a more plethoric and vigorous frame; and in such cases the debility induced by the use of the lancet, however imperatively it might have been demanded, is very apt to bring on hysteria. We state this opinion from observation.

We must refer to the work itself for several diagnostic marks which tend to distinguish the local affections above referred to from true inflammatory attacks. We cannot, however, dismiss this part of the subject without observing that, in the remarks which Dr. Hall offers upon it, we discover clear proofs of an observant and intelligent mind.

Part II. *Of some of the diseases incident to the puerperal state.*—This class of diseases may be considered as embracing all those morbid affections which arise out of the state of pregnancy, of child-bearing, or of lactation. The object of the present section is to treat only of certain morbid affections which occur in the puerperal state, and which appear to the author not to have received the degree of consideration which is due to them. Many interesting and practical observations are made upon "puerperal inflammation within the abdomen," upon "stomachal and intestinal irritation," and upon the effects of "loss of blood." We have already referred to the latter subjects in our review of the author's "Medical Essays," in our December Number. They are, in the present volume, treated in a more comprehensive manner, and merit particular attention.

In our notice of Dr. Hall's former publication, we deferred giving an abstract of his opinions upon "the morbid effects of undue suckling," as we were then promised a more matured consideration of the subject, which in the present volume is treated of in the third Chapter of the third Part. "The first effect of undue suckling is general debility of the whole system. There is soon a defective state of sanguification and nutrition, and of the nervous powers, inducing paleness, thinness, and nervousness. The stomach soon becomes enfeebled, and unable to bear the necessary food; the bowels become constipated, flatulent, and apt to be affected with diarrhœa. As further consequences, the head, the heart, and the lungs suffer, and there are various morbid affections of these organs." The general symptoms of debility increasing, "the patient is apt to try to support her strength by a generous diet and wine. But the effort is vain: the stomach is enfeebled, and consequently unable to bear the increased burthen imposed upon it."

The term "undue lactation" is indefinite. The constitutional powers of one woman will enable her to suckle her infant for a considerable time, not only without inconvenience, but with real advantage; whilst, in a less vigorous frame, general debility may be very speedily induced, or perhaps even the attempt to suckle at all may be injudicious and imprudent. We must observe, however, that such is the benevolent provision of nature, that few women are incapable of suckling their offspring, although, particularly in fashionable life, many are disinclined to fulfil this important moral obligation. Undue lactation, then, is not necessarily protracted lactation.

Amongst the first effects (according to Dr. Hall) of undue lactation, "is a disordered state of the alimentary canal." His treatment is modified accordingly.

"The effects of this state of the bowels are frequently experienced in the affection of the head, combining pain, vertigo, or a sense of weight, with intolerance of light, perhaps, but more frequently of sound. This affection of the head is most effectually relieved,—not by blood-letting, not by active purgatives, not by abstinence,—but by carefully correcting the disorder, and regulating the evacuations, of the stomach and bowels; whilst we enjoin a mild diet administered frequently, great quiet, and the strictest exemption from fatigue, hurry, or disturbance.

"From all this it would appear that this affection does not consist in mere fulness of the blood-vessels of the brain: it is, however, more than probable that the due balance of the circulation is not preserved in the arterial and venous systems; and it has already been observed in this work, that a state of general exhaustion is not incompatible with congestion of the brain. Leeches and cupping, cold lotions to the head, and blisters to the nape of the neck, are therefore often of the greatest service; only they must be employed with a due regard to the exhausted state of the system at large.

"When the head is affected more by the state of exhaustion than by the disordered condition of the alimentary canal, the symptoms are vertigo, a sense of pressure, and occasional faintishness, rather than throbbing and intolerance of light; and, in the more protracted cases, the chest is frequently more affected than the head. It is important to repeat, that, even in this case, the state of the circulation within the head is so far deranged, that paralysis has occurred during the state of exhaustion, as apoplexy has occurred in the exhaustion from loss of blood; and it has already been observed that undue lactation sometimes leads to mania.

"That the affection of the head ascribed to the influence of a loaded state of the stomach and bowels, with exhaustion, does indeed depend upon that cause, is proved, I think, by the mode of treatment usually found to be most efficacious, but especially by the fact that such a state of affection of the head is sometimes immediately produced on the patient's taking indigestible food. In one case the attack was almost instantly produced by eating pork; and many such facts must be known to every experienced physician." (P. 337.)

The fourth Chapter contains a few brief observations upon the "effects of Menorrhagia and Leucorrhœa," which are very similar to those arising from undue lactation.

The volume concludes with some remarks upon the decline of the vital powers in old age.

Although the present work is occupied in a great measure by the consideration of subjects upon which Dr. Hall has previously offered his opinions to the profession, it is well worth an attentive perusal. His views of the nature and treatment of the different diseases upon which he treats are expounded at greater length, and are occasionally illustrated by appropriate cases.

---

*A Series of Engravings intended to illustrate the Structure of the Brain and Spinal Chord in Man.* By Herbert Mayo, Surgeon, and Lecturer on Anatomy.—Burgess and Hill, London, 1827.

The School of Great Windmill-street has been rendered remarkable both by the great reputation of its founder, and by the succession of eminent men who have delivered anatomic lectures in its theatre. The names of William and John Hunter, Cruickshank, Baillie, and Wilson, are associated with the most important improvements which have of late years been made in anatomy, physiology, medicine, and surgery. Of those who now fill their places, it would not become us to speak in terms of such direct commendation; but their writings are before the profession, and render this unnecessary. The work to which we have at present to call the attention of our readers is one of a kind never attempted but by those who have the improvement of science zealously at heart: the expence attending their publication is great, and the price seldom such as to afford any adequate return.

There are three different methods of studying the brain. First, we may examine it externally; secondly, we may divide it by transverse slices; thirdly, we may endeavour to trace the internal structure of the parts, by as it were unravelling them.

The first of these methods is extremely well illustrated in GORDON's System of Anatomy; and it is astonishing how extensive a knowledge of the brain may be obtained by merely scrutinising it externally, and separating the parts where there are fissures, so as to look in upon the various cavities or ventricles as far as can be done without laceration.

The second method, or that of slicing, is the one most frequently employed, and most applicable for pathological purposes, as it is scarcely possible that any considerable portion of diseased structure should pass unnoticed. This mode of examining the brain is of more value to the student after than before he has practised the others; it enables a person skilled in dissection, to display the condition of the different parts with the least possible disturbance or alteration of their appearance.

The third method, or that of tracing the distribution of the structures, is impracticable in the recent brain. VIEUSSENS and MALPIGHI adopted the plan of boiling the brain in oil, and appear to have pursued their investigations to a considerable extent; but REIL was the first who duly appreciated the advantages of this method of examination. The others ascertained that the brain contained fibres, and even knew something of their distribution;* but Reil, having hardened the brain in alcohol, made this a particular study, and published a succession of papers, the first of which appeared in GREN's Journal in 1795. "He details the whole fibrous structure of the cerebellum; traces with great minuteness the bands of fibres, from the corpora pyramidalia through the annular protuberance to the peduncles of the brain proper, and thence through the corpora striata to the convolutions; follows carefully and precisely the fibres of the corpus callosum, and of the anterior commissure, to their destination in the hemispheres; describes the structure of

* See MALPIGHI's Exercitatio Epistolica de Cerebro, 1664.

the convolutions with considerable accuracy, and alludes briefly to other parts. In conclusion, he observes that, if one were to fix upon a point in the nervous system, such as the medulla oblongata, this system might be regarded as radiating from this point, to the extremities of the nerves on the one hand, and to the extremities of the fibres in the cerebrum and cerebellum on the other; or, reversing the matter, all these nerves and fibres might be considered as converging from their extremities towards the medulla oblongata."

This information, however, as stated above, is scattered through separate essays, without being constructed into a general view of the whole. The subject was afterwards taken up by Drs. Gall and Spurzheim, who adopted the views of Reil, and to the persevering manner in which they have dwelt upon them, and to the peculiarity of their phrenological doctrines, (though not necessarily connected with the anatomical details,) it is that this last method of studying the structure of the brain has become so much in vogue of late years. At the same time we repeat what we have on more than one occasion stated before, that whoever takes the trouble to consult the writings of Vieussens, Malpighi, Willis, Reil, and Mayer, will find few instances in which he can attribute originality to what is correct in the anatomical part of the works of Drs. Gall and Spurzheim.

To give a comprehensive view of the discoveries of the anatomists above mentioned, with the addition of some original observations of his own, appears to have been Mr. Mayo's object in the present volume. It consists of drawings from actual dissections, with copious references, in the manner adopted by Soemmering, in his fasciculi on the Organs of the Senses.

The first plate represents the Spinal Chord, (of which, by the bye, we believe, no account is given by Reil.) As a mere description of plates would at best be uninteresting, and perhaps not easily rendered intelligible, we shall therefore only touch upon those parts which are either new or refer to disputed points.

We were surprised to find it asserted by Magendie and Desmoulins, in their recent work on the Nervous System, that the bundles of fibres of the anterior pyramid do not cross, but merely meet and continue their course in juxta-position. Their words are—"Mais de même que les fibres d'une de ces pyramides superieures ne se croisent pas avec celles de l'autre, qu'elles ne font que se juxta-poser par intersection comme les lignes qui determinent les cotés d'un angle de même non plus *les fibres des pyramides inferieures ne se croisent pas*."* As the above quotation is in reference to some of the lower animals, the word *inferieur* corresponds to *anterior*, as applied to the human subject. Now, Mr. Mayo adheres to the former supposition, that there is an actual decussation; and shews that in addition the anterior pyramids are partly derived from the white matter of the same side. It is remarkable that the anterior pyramid on emerging from the pons Varolii is vastly larger than on entering it. Gall and Spurzheim conjectured that, in its transit, it received an addition by the union of fresh fibres. In the fifth figure of Plate I. we find this demonstrated by Mr. Mayo, who, by dissection, has shown the bundles which arise in the pons, and go to unite themselves to the crus cerebri,—i. e. to the anterior pyramid after it has passed the pons Varolii.

Another remarkable circumstance presents itself to our notice in the second Plate: it is the existence of a ventricle in the Pes Hippocampi. It will be remembered that the distribution of white and grey matter at this point gives somewhat the appearance of a *roll pudding*, the part being coiled up; and, when a section is made of it, the white and grey matters are seen to alternate. Now, at one spot, for no inconsiderable extent, these layers, although adjoining, do not adhere, as may be shown by slightly pressing them: in other words, a ventricle exists in this situation. Probably this may be connected with the function of the parts, as we find elsewhere that the presence of a ventricle is associated with increase of energy.

The general object of the second Plate is to represent alternations of colour in the medulla oblongata and pons Varolii: these delineations are taken from recent dissections, and we may observe, connected with them, what appears to have been a very singular mistake on the part of Dr. Gordon, of Edinburgh. This generally accurate anatomist asserts that, when a section is made of the annular protuberance and medulla oblongata, the colour of the alternate layers varies according to the direction in which they are divided, and even gives a plate in which the parts, being cut both vertically and horizontally, the matter which in the *horizontal* section is white, in the *vertical* section is grey,

* Anatomie des System Nerveaux des Animaux à Vertebres; par M. Magendie et Desmoulins.

and *vice versâ.* Mr. Mayo, on the contrary, looks upon the appearance as an optical deception; the white matter being always white, and the grey matter grey.

Plates III. to VI. represent the manner in which the white medullary matter is distributed through the brain, illustrating the general position that it is the medium of communication between the different parts of the grey matter, which last would appear to be the organ on which function is more immediately dependent. Perhaps we might compare the white matter to the nerves,—not essentially possessing functional power, but transmitting it so long as they remain continuous. These plates, which are beautifully executed, show very distinctly the different contrivances which, so to speak, are adopted to secure perfect intercourse between the various parts of the encephalon. Thus we have, first, white bands running from one convolution to that which is next it, and this throughout so as to form a continuous chain of communication; secondly, we have bands running between the more distant parts of the same side, securing, as it were, a less circuitous connexion; and thirdly, we have bands running transversely, by which the two hemispheres are enabled to co-operate: while in addition we have the fibres which extend from the cerebrum to the cerebellum, and from both to the medulla oblongata.

Plate VII. represents the origin of the Nerves. Here also we observe a difference between Mr. Mayo and M. Magendie: the latter represents the sixth nerve as running down along the anterior pyramid to take its origin from the front of the spinal marrow; the former, as running back by the lower edge of the pons Varolii, to arise from the back part of the medulla oblongata.

It is, of course, obviously impossible to convey an idea of the effect of plates by mere verbal description, but, as we before said, they are admirably executed, and afford a valuable addition to the library of the anatomist. We understand that the facts which they represent are exhibited by Mr. Mayo in each anatomical course given at the School of Great Windmill-street.

*Répertoire général d'Anatomie et de Physiologie, Pathologiques, et de Clinique Chirurgicale; ou, Recueil de Mémoires et d'Observations sur la Chirurgie, et sur l'Anatomie et la Physiologie, considerées dans les tissus sains et les tissus malades.* Tome II. No. IV.—Paris, 1827.

The present Number of the Repertoire is not, perhaps, so rich in practical information as its predecessors. We shall give the substance of the most interesting papers.

The two first papers are by M. Lallemand and M. Breschet, upon the subject of *certain Sanguineous Tumors of an equivocal character, which appear to be aneurisms of the arteries of the bones.* This disease has only been witnessed in young persons and in adults. It arises spontaneously in most cases; sometimes, however, it follows blows, or any other external violence. It occasionally succeeds to gouty or rheumatic affections of the knee.

*Symptoms.*—The attack is sudden, and without any known cause in many instances. The disease is usually situated at the posterior part of the leg, below the knee, and affects the tibia or fibula, and perhaps both of these bones simultaneously. The tumor is painful; the veins of the whole limb are swoln and distended, and varicose. Pain, and a purplish blush, occupy the limb throughout its whole extent. A pulsation is soon perceptible, which at first is deep-seated. It is synchronous with that of the arteries, and without any noise, (*bruissement.*) The pulsation ceases entirely if the artery is compressed between the heart and the tumor. The patient complains of constant pain in the part. Motion of the limb, or of the knee alone, is impeded, and more or less painful. Pressure of the tumor with the finger produces upon some parts of it a sound resembling the rumpling of parchment, or still more the breaking of an egg; whilst in other points of the swelling considerable pressure gives rise to no such sound.

*Structure of the tumor.*—Dissection of the parts affected, after the amputation of the limb, has invariably shown the principal vessels, throughout their whole extent, of perfect integrity. They may be injected or dissected without presenting any solution of continuity. But this is not the case with the small vessels which penetrate the spongy substance of the bones. The bony tissue has always been found affected; and, according to the progress of the disease, it is found that the alteration of the bone has proceeded from the internal to the external parts. The cellular tissue of the bones is either in great part or wholly destroyed; the cavity of the bone is enlarged, filled with coagulated blood, disposed in concentric layers, as in old aneurismal tumors; and these clots form one or several foci, each communicating with an arterial branch. The external table of the bone still remains, but thinner than natural, and destroyed in several of its parts, and offering but a feeble resistance in comparison with a cartilaginous surface, which yields to the finger, but quickly recovers itself. Sometimes the bone may be crushed easily, like an egg-shell. The periosteum, and the aponeurotic expansion, are generally thicker and firmer than in the healthy state, and sometimes they pass into a fibro-cartilaginous state. The articulation near the seat of the disease has always been found healthy, even when it was only separated from the seat of the malady by an expansion of cartilage. Injection has proved that the vessels of the limb are healthy, but that the arteries supplying the substance of the bone are increased in volume, and that they passed from the centre of the bone into the aneurismal sac by several orifices.

*Treatment*.—Amputation has enabled us to study and to detect the nature of the disease by dissection of the parts affected; and the ligature of the principal trunk, by cutting short the malady, has left no doubt upon the subject, and proves demonstratively the aneurismal character of this species of tumor. If, indeed, the affection depended upon the bones,—if it was an osteo-sarcoma, a fungus of the periosteum, or a varicose tumor, the ligature of the principal arterial trunk could have but little influence, either upon its progress or termination.

We are indebted to the clinical instruction at the Hôtel Dieu for this information; and M. Dupuytren may claim the merit of having detected the nature of the disease, and also of having first suggested the best mode of treatment. In M. Breschet's opinion, this affection may be compared to erectile tumors of the soft parts. "John Bell, and subsequently to him all his countrymen, have assigned to it the name of aneurism by anastomosis. This term is improper, and does not accord with the anatomical nature of the disease. The pulsation observed in all these tumors, which is sufficiently powerful to have led to the comparison between them and aneurisms properly so called, results from the synchronous movements of dilatation and contraction of all the small arteries which pass from the bony substance to the part affected. From these partial but simultaneous movements results a continued motion. This also has been frequently observed in erectile tumors of the lips, and of the globe of the eye, &c."

Is this disease, in which we find the arteries of the bones and the bony tissue itself affected primarily, seated in the small arteries of the bones, or in the osseous substance itself? If, on the one hand, it is considered that the disease is arrested by the application of a ligature to the principal arterial trunk, it may be presumed that the original and chief affection is in the vascular system. But is there not reason to conclude, from the healthy state of the vascular branches, which give origin to those which penetrate the bones, and in the return of the disease after the ligature, as well as from the deep-seated alteration of the osseous substance, that there is an original disease of the bones? Upon this part of the subject, then, some doubt still remains, which future investigations may resolve.

M. Breschet afterwards proceeds to point out the distinctive characters between the disease just described and affections of the bones themselves.

Several cases also are related of aneurism of the arteries of the cellular tissue of the bones.

In the fourth section of the paper the following question is discussed: Is the application of the ligature to the principal vessel the best mode of affecting a cure; and is it to be had recourse to in every case?

In favour of the application of the ligature, M. Breschet not only has the analogy of other aneurismal affections, but also the success obtained from it in the cases he has related. Neither topical applications nor compression can be serviceable in such cases, whatever may be their utility in other aneurismal swellings. The ligature, then, is the proper remedy. If, however, there is a deep-seated and considerable affection of the substance of the bones, amputation becomes the only resource.

From the facts and arguments adduced, M. Breschet concludes that—

1st. The arteries of the bones may be affected with aneurism, like the vessels of the soft parts.

2d. Aneurisms of the osseous tissue present several symptoms resembling aneurisms, properly so called.

3d. This disease, which has not hitherto been described, differs from all other affections of the bones, and from fungous tumors of the periosteum, as well as from accidental sanguineous fungous tumors.

A case of *Wound of the Heart* is related by M. Ferrus, in which the instrument which inflicted the injury remained in the heart. The patient survived about three weeks. Several similar cases have been related by various authorities.

A successful case of the *Application of the Ligature to the external Iliac Artery, in a Case of Aneurism,* is also recorded. It was read by M. DUPUYTREN, who performed the operation, at the Academy of Sciences, in July 1819.

---

*The Hunterian Oration, delivered before the Royal College of Surgeons in London, on Wednesday, February* 14, 1827. By H. LEIGH THOMAS, F.R.S. Member of the Imperial Academy of St. Petersburg, &c.—4to. pp. 28. London, 1827.

To say that the name of HUNTER is to be most highly honoured, is but to echo the universal sentiment of all who know how much science in general, and our profession in particular, owe to his labours: yet we question the wisdom of offering an annual tribute of praise to his memory. Eulogy which is methodically set about as a matter of business, comes not warm from the heart, and is listened to with indifference,—or, at least, without that sympathy of feeling which pervades alike the speaker and his hearers, when spontaneously, and perhaps without premeditation, he paints the character of a friend in the glowing colours of reverence and affection.

We do not make these remarks as applicable to the Oration before us in particular, but to all such in general. Associated with HUNTER, Mr. THOMAS naturally refers to CRUICKSHANK; and, as his connexion with him was of a kind to afford the best opportunities of becoming acquainted with his character, we shall extract the account which he gives of this distinguished anatomist, conceiving that the dispositions, manners, and habits of men who have been eminent in the profession must prove interesting and instructive to our readers.

"To the few friends who now survive him, I do not fear to appeal for the truth of my assertion: that he was a man of more than ordinary stamp; one whose talents, and numerous excellences, inspired a strong and devoted attachment; whose virtues were warm from the heart; whose errors were from the head alone, and so mingled with and allied to estimable qualities, as only to excite a more lively interest in his welfare.

"Of his talents, the works which he has left behind him are the best testimony: of his book on the Absorbents there can be but one opinion, that it classes with the most valuable medical publications of modern times; but it was those alone who enjoyed the advantages of his friendship and the pleasure of his private society, who could fully appreciate his worth, or judge from what he was what he might have been.

"No one could have been more perfectly disinterested, or less mercenary and selfish; nor have entertained a loftier disdain for every species of artifice and deception.

"His door was never closed against sickness or sorrow: nay, almost fortunate might those poor persons consider themselves who, labouring under any new or extraordinary diseases, applied for his assistance: in such cases his devotion to his profession, his keen and inquisitive pursuit of knowledge, and his natural kindness of heart, made his time, his talents, and his purse equally at their service.

"Diseases will, of course, occasionally present themselves, in which all human aid is vain; but it may be truly asserted of him, that, though his skill necessarily failed in such cases, his benevolence never did.

"Though sometimes irascible from hurry of professional duties, his manners with his patients were usually conciliating and kind: for an example, I may quote the authority of Dr. Samuel Johnson, who, in his last illness, grateful for his kind and assiduous attendance, remarked to a mutual friend, that 'Cruikshank might justly be called, in the phrase of his country, a sweet blooded man.'

"He was in habits of intimacy with most of the literary men of his time; by whom he was not more valued for his great medical skill, than for his taste in literature and the arts, and acquirements in general knowledge.

"To these he joined a tenacity of memory as valuable as it was rare; which, with his correct judgment, gave a readiness and aptitude of quotation, that threw a peculiar charm over his conversation.

"How much it is to be regretted that great talents and genius are so frequently accompanied by such a morbid susceptibility of nerve, as casts a gloom over the days they were destined to brighten; that the close and intense application, the sedentary habits, by which superiority in most sciences is acquired, should engender a disease which blights the fairest prospects; making those retreat who are best qualified to advance; and those to distrust who ought to have most confidence in themselves!

"This is the more to be lamented, as this physical infirmity is but too apt to foster the moral one,—a suspicious temper: a heavier misfortune cannot afflict human nature; pervading alike public and private life, and under the influence of which the best-intentioned actions are misconstrued—affections alienated—friendships dissolved!

"Labouring under this nervous malady, which had assailed him from early youth, Mr. Cruikshank's pre-eminent talents, and many noble qualities, were occasionally obscured, and their course impeded. To the same cause may in a great measure be attributed his want of success as an operative surgeon. The head had every requisite for the most difficult operations, but the tremulous hand failed often in the execution of them. This, of course, prevented his elevation to that rank in the profession to which his education and abilities so fully entitled him.

"His celebrated preceptor, Dr. Hunter, having made the department of midwifery the more immediate object of his practice, it was naturally to be expected that the pupil would follow in the same path. But still his superior knowledge of anatomy often led to his being consulted in cases which may be considered as more properly belonging to the province of the physician.

"Forty years back this was accounted a professional innovation not to be tolerated, and of course excited much general animosity against him.

"Highly independent of spirit, and very tenacious of a just appreciation of his talents;—dignified and fearless by nature, but timid through abstraction of mind, and shrinking through acuteness of feeling, his was a character as little calculated to struggle against those hostile feelings, as to employ those conciliatory measures by which they might have been appeased.

"With talents that would have honoured any profession, and acquirements that would have embellished a high private station, there could not have been a man more unfit for mingling in the common intercourse of active life; or one more deficient in that knowledge of the world which should regulate the judgment, and which can alone preserve us from inconsistency."

---

*Observations on the Impropriety of Men being employed in the Business of Midwifery.*—Hunt and Clarke, London, 1827.

We have looked over this pamphlet with mingled feelings of pity and disgust,—pity for the credulity of its author, disgust for the sentiments it contains. Its object is to assert—for there are no proofs—that the "lusts" of accoucheurs is "equally powerful and inextinguishable," and, as a necessary consequence, that they are constantly employed in debauching their patients; so that "the minds of more women are corrupted through their arts than by those of all other classes of men combined."

Such of our readers as have not seen this precious production will scarcely credit what monstrous absurdities it contains; but we shall give one or two specimens, which will render comment unnecessary. The whole fabric rests upon three propositions, which are laid down in great form; they are as follow:

"1st. Lust is the most powerful of all the appetites: to whatever extent it may be gratified, its demands are soon again renewed, especially if attracted by variety in its object; and, when the body has lost its power of indulgence, the mind frequently retains its desires, sometimes even heightened in a great degree. Therefore, men advanced in years, when inclined by their vicious propensities, are empowered by their experience, and consequent subtleties, to contaminate the minds of women more than younger and less experienced men. Lust being thus the most powerful of all the appetites, it is (and it is necessary for natural purposes that it should be) less under the influence of the reason than any other appetite; for, if we had the power of coolly deliberating upon the effects of indulgence, the intentions of nature would be often frustrated. In consequence whereof, this appetite is most subject to abuse.

"2d. It is natural to man to abuse power and opportunity.

"3d. From the peculiar nature of their profession, accoucheurs have the greatest incitement to lust, and possess more ready means and pretences by which they may gratify it, to a great extent, than any other class of men." (P. 8.)

In the next page we have a quotation from Scripture—that "it is easier for a camel to go through the eye of a needle than for a rich man to enter the kingdom of God;" and then forsooth the author likens the accoucheur to the camel!

But it is not to the practice of midwifery that these abuses are confined: we find that—

"When a young medical man has acquired a knowledge of the rudiments of his profession, he is introduced at one of the London hospitals to complete his studies; and he cannot finish his education more advantageously, as there can be no scenes so subversive of moral principle as those which are exhibited in the periodical inspections at those institutions. In the course of one morning's round, there are not less than fifty women, chiefly young ones, whose persons are exposed and examined in the grossest manner by one of the principal surgeons, in the presence of the pupils; who, in their turn, daily supply the place of the principal, by exposing and examining themselves the persons of these young women in a similar manner; and upon whom many of them, of course, frequently practise every description of licentiousness that the place and circumstances will admit of." (P. 11.)

We will not insult the understandings of our readers by refuting such palpable misrepresentations: we are utterly at a loss to conjecture where such scenes are to be met with, and indeed, but for the appearance of this pamphlet, we could not have supposed that there was any one capable of having his imagination so thoroughly polluted.

The rest of the pamphlet consists of mere gossip; for the author appears to have listened to every idle story, and, from

2

the collection with which he has favoured us, it is evident he has been practised upon by persons who have been laughing at him all the while.

We believe that the College of Surgeons means to require attendance on Midwifery Lectures of those applying for a diploma; and we earnestly recommend to the Apothecaries to reconsider their late decision against a similar regulation: they may be assured that the great majority of the profession, and the public, are of opinion that they who practise any branch of medicine should first undergo some ordeal, to show that they are qualified to do so.

